# Cavotricuspid isthmus ablation guided by force‐time integral – A randomized study

**DOI:** 10.1002/clc.23805

**Published:** 2022-03-17

**Authors:** Dimitrios Asvestas, Vasileios Sousonis, George Kotsovolis, Stavros Karanikas, Anastasia Xintarakou, Eleftherios Sakadakis, Angelos G. Rigopoulos, Andreas S. Kalogeropoulos, Panos Vardas, Stylianos Tzeis

**Affiliations:** ^1^ Department of Cardiology, Mitera Hospital Hygeia Group Athens Greece

**Keywords:** ablation, atrial flutter, cavotricuspid isthmus, force‐time integral

## Abstract

**Background:**

Force‐time integral (FTI) is an ablation marker of lesion quality and transmurality. A target FTI of 400 gram‐seconds (gs) has been shown to improve durability of pulmonary vein isolation, following atrial fibrillation ablation. However, relevant targets for cavotricuspid isthmus (CTI) ablation are lacking.

**Hypothesis:**

We sought to investigate whether CTI ablation with 600 gs FTI lesions is associated with reduced rate of transisthmus conduction recovery compared to 400 gs lesions.

**Methods:**

Fifty patients with CTI‐dependent flutter were randomized to ablation using 400 gs (FTI400 group, *n* = 26) or 600 gs FTI lesions (FTI600 group, *n* = 24). The study endpoint was spontaneous or adenosine‐mediated recovery of transisthmus conduction, after a 20‐min waiting period.

**Results:**

The study endpoint occurred in five patients (19.2%) in group FTI400 and in four patients (16.7%) in group FTI600, *p* = .81. First‐pass CTI block was similar in both groups (50% in FTI400 vs. 54.2% in FTI600, *p* = .77). There were no differences in the total number of lesions, total ablation time, procedure time and fluoroscopy duration between the two groups. There were no major complications in any group. In the total population, patients not achieving first‐pass CTI block had significantly higher rate of acute CTI conduction recovery, compared to those with first‐pass block (29.2% vs. 7.7% respectively, *p* = .048).

**Conclusions:**

CTI ablation using 600 gs FTI lesions is not associated with reduced spontaneous or adenosine‐mediated recurrence of transisthmus conduction, compared to 400 gs lesions.

## 1 INTRODUCTION

Cavotricuspid isthmus (CTI)‐dependent atrial flutter (AFL) is a common arrhythmia caused by a macroreentrant circuit rotating along the tricuspid annulus. Catheter ablation, achieved by creating a line of bidirectional conduction block across the CTI, is considered the treatment of choice for CTI‐dependent AFL.^1^ Based on the recently issued ESC guidelines, catheter ablation is recommended not only in patients with multiple symptomatic AFL recurrences, but it should be considered, as well, even after the first episode of symptomatic AFL.^2^ Considering the low threshold for invasive management of CTI‐dependent AFL, the proposal of specific ablation guidelines to improve the durability of CTI bidirectional block has important clinical implications.

Deployment of effective ablation lesions is affected by several procedural factors, such as catheter stability, duration of energy delivery, temperature, impedance drop, and diminution of electrogram amplitude.^3^ Contact force (CF) is a marker of ablation lesion quality and transmurality.^4^ Low CF values are associated with ineffective lesion formation during atrial fibrillation (AF) ablation^5^ and specific CF recommendations have been issued to improve the durability of pulmonary vein isolation (PVI).^6^ Force‐time integral (FTI) incorporates the average CF (in grams) and duration (in seconds) during radiofrequency (RF) energy application and is a marker of ablation lesion size and volume at a given power setting.^7^ In the AF ablation setting, a minimum FTI of 400 gram‐seconds (gs) reduces the risk of pulmonary vein (PV) reconnection.^8^ However, specific FTI values to guide CTI ablation have not been proposed and the target limit of 400 gs is usually adopted, in everyday clinical practice. Thus, it remains elusive whether higher FTI values could result in reduced rates of CTI conduction recovery.

The aim of this randomized study was to test the hypothesis whether CTI bidirectional block achieved by sequential spot lesions with an FTI of 600 gs is associated with reduced rate of acute transisthmus conduction recovery, compared to 400 gs FTI lesions.

## 2 METHODS

### 2.1 Study protocol

This prospective, single‐center, randomized study enrolled 50 patients with symptomatic CTI‐dependent AFL, who underwent first‐time CTI ablation from August 2019 to December 2020. The study was approved by the Institutional Review Board of Mitera Hospital, Athens, Greece, and conformed to the ethical guidelines of the Declaration of Helsinki. Study participants provided written informed consent and all CTI ablation procedures were performed by two experienced electrophysiologists (prior experience of over 300 CTI ablations). Patients were randomized to two groups, using the sealed envelope method: in group FTI400, patients underwent CTI ablation with spot lesions of 400 gs, while, in group FTI600, patients underwent CTI ablation targeting 600 gs spot lesions. The study endpoint was defined as either spontaneous or adenosine‐induced recovery of transisthmus conduction, after a 20‐min waiting period.

### 2.2 Ablation procedure

Catheter ablation was performed with uninterrupted anticoagulation therapy. All patients received oral anticoagulants for at least 1 month before the procedure and antiarrhythmics were discontinued for at least 7 days. Venous access was obtained via the right femoral vein in all patients. Twelve‐lead electrocardiogram and intracardiac electrograms were recorded and analyzed using the LabSystem EP Recording System. A deflectable decapolar diagnostic catheter was placed in the coronary sinus and an open‐irrigated ablation catheter (TactiCath™ Quartz ablation catheter, Abbott, Inc.) was advanced into the right atrium. In patients presenting with ongoing AFL, standard entrainment maneuvers were performed from the CTI and the proximal bipole of the coronary sinus catheter to confirm the diagnosis of CTI‐dependent flutter. In patients presenting with sinus rhythm, preablation transisthmus conduction interval was measured in both directions (from the proximal coronary sinus to the lateral isthmus and vice versa). All cases were performed with the use of the NavX electroanatomical mapping system (St. Jude Medical).

**Figure 1:**
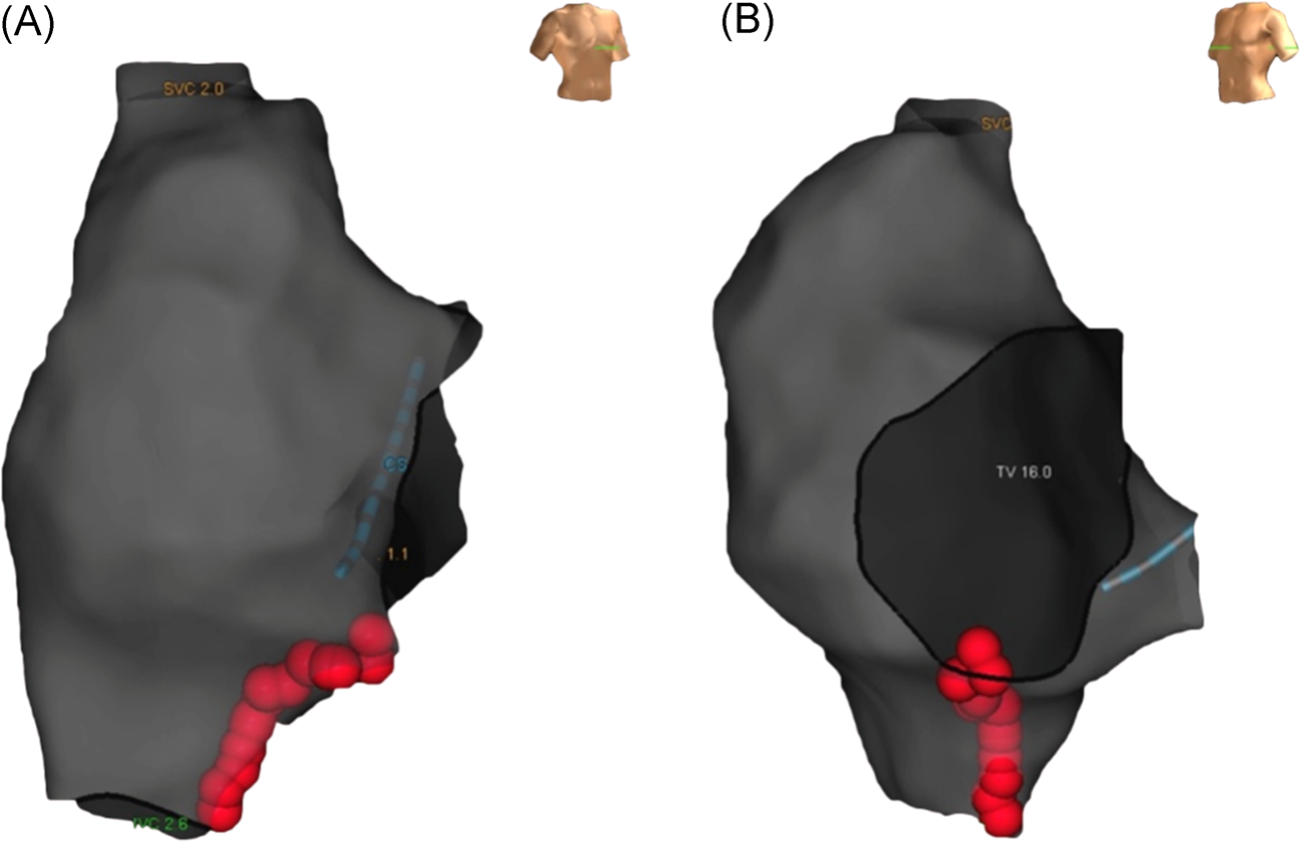
Ablation line along the cavotricuspid isthmus in a patient with typical atrial flutter in right anterior oblique (A) and left anterior oblique (B) views. Red dots represent ablation lesions with a force‐time integral of 600 gs

After advancing the ablation catheter to the tricuspid valve annulus, a CTI ablation line was performed by deploying sequential spot lesions, while gently withdrawing the catheter along the central isthmus to the inferior vena cava, after every lesion completion (Figure 1). Ablation was performed at a power of 30 W, using an irrigation rate of 17 ml/min. For every lesion deployed, a CF of 10–30 g was targeted. Lesion formation was guided by a target FTI value of either 400 or 600 gs in the two randomized groups, respectively.

The procedural ablation endpoint was the achievement of bidirectional CTI block, confirmed by differential pacing maneuvers,^9^ the presence of double potentials along the ablation line and the assessment of prolongation of the transisthmus interval in both clockwise and counterclockwise directions, during pacing from the coronary sinus ostium and the low lateral right atrium, respectively.^10^ First‐pass CTI block was defined as achievement of bidirectional CTI block upon the completion of the initially deployed CTI line, without the need for additional RF lesions.

**Figure 2:**
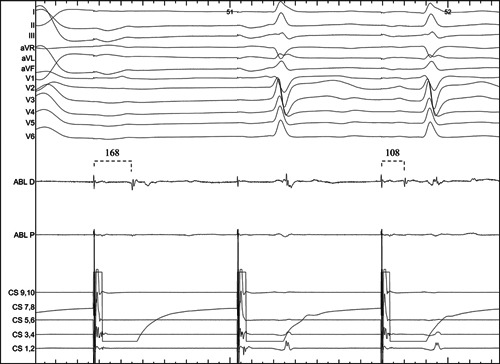
Transisthmus conduction recovery documented by shortening of the transisthmus interval (from 168 to 108 ms) during pacing from the proximal coronary sinus after adenosine infusion with associated transient atrioventricular block. ABL D: distal bipole of the ablation catheter; ABL P: proximal bipole of the ablation catheter; CS 9,10: proximal bipole of the coronary sinus catheter

Twenty minutes after documentation of bidirectional CTI block, the ablation line was assessed for spontaneous recovery of CTI conduction, using the same electrophysiological criteria as described above. In the absence of spontaneous recovery, a provocative test with intravenous adenosine was performed to evaluate the presence of dormant CTI conduction. Specifically, 6 mg of adenosine were rapidly infused intravenously, aiming to achieve at least one blocked P wave or a sinus pause of 3 s. In case of inability to achieve transient atrioventricular block or sinus pause, the infused dose of adenosine was increased by increments of 6 mg. Testing was initially performed under pacing from the proximal coronary sinus, with the ablation catheter positioned just laterally to the ablation line. The same testing was performed during pacing from the ablation catheter just laterally to the CTI ablation line. Dormant recovery of transisthmus conduction was defined as transient or permanent shortening of the transisthmus interval, following adenosine infusion (Figure 2). In case of adenosine‐mediated recovery of conduction, additional lesions were deployed until adenosine testing was rendered negative.

In every procedure, the following parameters were recorded: transisthmus conduction interval in both directions before and after CTI block, interval between double potentials along the ablation line, total number of RF lesions, total procedure and RF ablation time, total fluoroscopy duration and mean value of lesion size index (LSI) of all deployed ablation lesions.

### 2.3 Statistical analysis

Continuous data are presented as mean ± standard deviation, while categorical data as count and percentages. Pearson's *Χ* ^2^ test for categorical and Student's *t*‐test for continuous variables were used to compare parameters between the two groups. Tests were considered statistically significant if the *p* value was less than .05. Statistical analyses were performed with SPSS statistical software (version 16.0, SPSS).

## 3 RESULTS

### 3.1 Baseline characteristics

A total of 50 patients (70% males, age 63 ± 12 years) were enrolled in the study (26 patients in the FTI400 group and 24 patients in the FTI600 group). Patient baseline characteristics are reported in Table 1. Prior antiarrhythmic drug therapy had been administered in 15 patients in group FTI400 (Ic agents in 13 and amiodarone in 2 patients) and in 17 patients in group FTI600 (Ic agents in 13, sotalol in 1, and amiodarone in 3 patients).

**Table 1:** Baseline characteristics

Baseline characteristic	Group FTI400 (*n* = 26)	Group FTI600 (*n* = 24)	*p*
Age (years)	66.5 ± 10.5	60.0 ± 11.9	.05
Male gender	16 (61.5%)	19 (79.2%)	.17
BMI (kg/m^2^)	28.8 ± 3.6	29.2 ± 3.9	.76
LA diameter (mm)	40.3 ± 2.3	40.7 ± 1.9	.78
LVEF (%)	57.7 ± 6.7	59.4 ± 6.3	.38
CHA_2_DS_2_VASc score	2.0 ± 1.0	1.2 ± 1.1	.02
Prior AAD treatment	15 (57.7%)	17 (70.8%)	.33

Abbreviations: AAD, antiarrhythmic drug; BMI, body mass index; LA, left atrium; LVEF, left ventricular ejection fraction.

### 3.2 Procedural characteristics

Procedural characteristics of both groups are presented in Table 2. Transisthmus interval pre‐ and post‐CTI ablation and interval between double potentials post‐CTI block did not differ between groups. Moreover, postablation transisthmus interval was similar in patients showing spontaneous or adenosine‐mediated CTI conduction recovery, compared to those who maintained bidirectional CTI block during the 20‐min waiting period and adenosine provocation testing (proximal CS to lateral isthmus: 178.5 ± 46.3 ms vs. 170.7 ± 30.7 ms, respectively, *p* = .55; lateral isthmus to proximal CS: 179.6 ± 46.4 ms vs. 172.3 ± 30.2 ms, respectively, *p* = .57).

**Table 2:** Comparison of procedural characteristics between groups

Procedural characteristic	Group FTI400 (*n* = 26)	Group FTI600 (*n* = 24)	*p*
Preablation transisthmus interval (proximal CS to lateral isthmus, ms)	67.7 ± 23.8	63.4 ± 17.5	.52
Preablation transisthmus interval (lateral isthmus to proximal CS, ms)	70.3 ± 27.0	64.1 ± 15.1	.36
Postablation transisthmus interval (proximal CS to lateral isthmus, ms)	175.5 ± 37.8	168.0 ± 27.6	.44
Postablation transisthmus interval (lateral isthmus to proximal CS, ms)	175.4 ± 37.1	171.4 ± 28.2	.67
DP interval (ms)	109.7 ± 18.9	109.6 ± 17.4	.99
Adenosine dose (mg)	10.4 ± 3.2	10.5 ± 3.3	.95
Total ablation time (s)	642.5 ± 436.8	838.6 ± 1061.7	.4
Total RF lesions	15.5 ± 10.0	12.0 ± 7.6	.18
Procedure time (min)	77.8 ± 14.5	73.9 ± 17.4	.4
First‐pass block	13 (50%)	13 (54.2%)	.77
Fluoroscopy time (min)	4.9 ± 2.7	4.6 ± 2.8	.74
Mean LSI value	5.1 ± 0.4	5.5 ± 0.5	<.01

*Note*: Continuous data are presented as mean ± standard deviation, while categorical data as count with respective percentages provided in brackets.

Abbreviations: CS, coronary sinus; CTI, cavotricuspid isthmus; DP interval, interval between double potentials after cavotricuspid isthmus block; LSI, lesion size index; RF, radiofrequency.

Bidirectional CTI block was achieved in 49 of 50 patients. In one patient in group FTI400, bidirectional CTI block was not achieved and the patient was considered to have met the study endpoint. The mean LSI value was significantly higher in the FTI600 group, as compared to the FTI400 group (5.5 ± 0.5 vs. 5.1 ± 0.4, respectively, *p* < .01). Total number of RF lesions (15.5 ± 10.0 in group FTI400 vs. 12.0 ± 7.6 in group FTI600, *p* = .18), total ablation time (642.5 ± 436.8 s in group FTI400 vs. 838.6 ± 1061.7 s in group FTI600, *p* = .4) and procedure duration (77.8 ± 14.5 min in group FTI 400 vs. 73.9 ± 17.4 min in group FTI 600, *p* = .4) did not differ between compared groups. Targeting an FTI of 600 gs was associated with similar rates of first‐pass CTI conduction block compared to 400 gs (54.2% vs. 50%, respectively, *p* = .77). Fluoroscopy time was similar in both groups (4.9 ± 2.7 min in group FTI400 compared to 4.6 ± 2.8 min in group FTI600, *p* = .74).

There were no major procedural complications in any group. In group FTI400 one patient presented a minor groin hematoma and one patient a small pericardial effusion (<5 mm), without associated need for hospitalization prolongation.

### 3.3 Recovery of transisthmus conduction

The occurrence of the study endpoint (spontaneous or adenosine‐induced recovery of transisthmus conduction) was similar in groups FTI400 and FTI600 (19.2% vs. 16.7%, *p* = .81). In group FTI400, one patient failed to achieve bidirectional CTI block and was considered to have met the study endpoint; two patients showed spontaneous recovery of CTI conduction during the 20‐min waiting period and two patients after adenosine administration. In group FTI600, recovery of CTI conduction was documented in three patients spontaneously and in one patient following adenosine administration. In the total population, patients with first‐pass CTI block were less likely to show spontaneous or adenosine‐mediated recovery of CTI conduction, compared to those requiring additional ablation lesions (7.7% vs. 29.2%, respectively, *p* = .048).

## 4 DISCUSSION

To the best of our knowledge, this is the first study to compare prospectively two different target FTI values during CTI ablation. The key findings of our study are: (1) CTI ablation using spot lesions with an FTI of 600 gs is not associated with reduced rates of acute transisthmus conduction recovery compared to 400 gs lesions; (2) the rates of first‐pass CTI block were similar in both FTI groups, with patients failing to achieve first‐pass block being more likely to show acute recovery of CTI conduction; and (3) the use of a 600 gs target FTI did not reduce the number of ablation lesions, the total ablation and fluoroscopy time or the total procedure duration.

CTI catheter ablation is a safe and highly effective procedure for the treatment of typical AFL.^1^ However, recovery of transisthmus conduction may occur in a sizeable percentage of patients, resulting in arrhythmia recurrences.^11^ Therefore, achievement of durable lesions during CTI ablation has profound clinical implications. Catheter‐tissue CF, as well as the FTI are major determinants of RF lesion size.^4^, ^7^ The introduction of CF‐sensing catheters has enabled real‐time monitoring of these parameters, thus facilitating better contact during lesion deployment and optimizing ablation efficacy.^4^ In AF ablation, lower FTI values are associated with higher rates of acute PV reconnection and dormant conduction.^12^ Squara et al.^13^ have suggested a target FTI higher than 392 gs as predictor of lesion transmurality using bipolar electrogram criteria. Based on the findings of the EFFICAS I study, a minimum FTI of 400 gs is recommended to prevent gap formation and achieve durable isolation of the pulmonary veins.^5^ Incorporation of this FTI target value in the EFFICAS II study resulted in more durable PVI in catheter ablation of paroxysmal AF.^8^

Acute recovery of CTI conduction after a waiting period, following the documentation of bidirectional CTI block, is related to higher AFL recurrence rate in the long term.^14^ Additionally, adenosine administration can help unmask dormant transisthmus conduction, known to precede recovery of permanent CTI conduction.^15^ In our study, we incorporated these two approaches to compare the acute procedural efficacy of CTI ablation using two different target FTI values during sequential spot lesion deployment. Given that central CTI thickness is similar to that of the left atrium,^16^ in the first group we used the FTI target of 400 gs, which in the EFFICAS II study was shown to result in durable PVI.^8^ Since FTI predicts lesion volume and depth,^7^ we used a higher FTI target value (600 gs) in the second group to test whether this would lead to lower rates of acute CTI conduction recovery. Based on our results, spot lesions administered in the 600 gs FTI group failed to create a more durable CTI ablation line, as indicated by the similar rates of acute conduction recovery in the two study groups. This finding may suggest that lesions applied with FTI values of 400 gs already achieve transmurality.

In comparison to conventional CTI ablation without the use of CF‐sensing catheters, real‐time, CF‐guided AFL ablation is associated with shorter ablation time to achieve CTI block, along with a significant reduction in the number of lesions and RF energy delivery.^17^ Based on our results, incorporation of a target FTI of 600 gs, instead of 400 gs, during AFL ablation with a CF‐sensing catheter did not significantly reduce the number of ablation lesions, procedure duration, or the total ablation and fluoroscopy time.

First‐pass isolation of the pulmonary veins during AF ablation is associated with lower reconnection rates^18^ and deployment of a meticulous ablation lesion set at the first attempt is associated with durable electrical isolation. Similarly, Viola et al.^19^ have recently demonstrated that first‐pass CTI block constitutes a strong predictor of a durable isthmus ablation line formation. In the same context, we showed that, in the total population, patients presenting first‐pass CTI block were less likely to show spontaneous or adenosine‐mediated recovery of CTI conduction compared to those requiring additional ablation lesions to achieve CTI block. It is worth noting that patients ablated with an FTI of 600 gs failed to show higher rates of first‐pass block, which is in line with the similar acute CTI conduction recovery rates observed in both groups in our study.

In our study, adoption of a higher target FTI of 600 gs did not result in increased rate of procedural complications, compared to an FTI of 400 gs. Interestingly, Gould et al.^20^ have used an FTI as high as 800 gs to guide CTI ablation without reporting any major complications.

## 5 LIMITATIONS

This study has several limitations. First, this is a single‐center study with a limited number of patients. Therefore, larger multicenter randomized studies are needed to confirm our results. Second, the study was designed to assess acute recovery of CTI conduction without reported data on patient follow‐up, regarding long‐term recurrence of AFL.

## 6 CONCLUSION

CTI ablation using spot lesions with an FTI of 600 gs is not associated with lower rates of spontaneous or adenosine‐mediated recovery of CTI conduction, compared to a target FTI of 400 gs. Our study does not support an incremental benefit from incorporating FTI values higher than 400 gs during CTI ablation, in terms of acute procedural efficacy.

## CONFLICTS OF INTEREST

The authors declare no conflicts of interest.

## Data Availability

Data available on request from the authors.
